# The SIRT-1/Nrf-2/HO-1 antioxidant defense axis in adult attention-deficit/hyperactivity disorder

**DOI:** 10.1007/s11011-026-01845-5

**Published:** 2026-04-21

**Authors:** Nilifer Gürbüzer, Alev Ozkaya, Filiz Mercantepe

**Affiliations:** 1 Department of Psychiatry, Erzurum Faculty of Medicine, University of Health Sciences, Erzurum, Türkiye; 2 Department of Biochemistry, Erzurum City Hospital, Erzurum, Türkiye; 3 Department of Endocrinology and Metabolism, Faculty of Medicine, Duzce University, 81620 Duzce, Türkiye

**Keywords:** Attention-Deficit/Hyperactivity Disorder, Oxidative Stress, SIRT-1, Nrf-2, HO-1, TNF-α

## Abstract

Attention-Deficit/Hyperactivity Disorder (ADHD) frequently persists into adulthood and is associated with substantial functional impairment. Beyond classical neurotransmitter dysregulation, increasing evidence points to oxidative stress and impaired cellular defense mechanisms as key contributors to ADHD pathophysiology. However, data on upstream redox-regulatory pathways in adult ADHD remain limited. To evaluate serum tumor necrosis factor-alpha (TNF-α), heme oxygenase-1 (HO-1), nuclear factor erythroid 2–related factor 2 (Nrf-2), and sirtuin-1 (SIRT-1) levels in adults with ADHD and to examine their associations with symptom severity and clinical features. This cross-sectional study included 60 adults diagnosed with ADHD and 60 age- and sex-matched healthy controls. ADHD diagnosis was established using the Structured Clinical Interview for DSM-5 Clinician Version (SCID-5/CV) and the DIVA 2.0 diagnostic interview. Symptom severity was assessed using the Adult ADHD Self-Report Scale (ASRS). Serum TNF-α, HO-1, Nrf-2, and SIRT-1 levels, along with routine laboratory parameters, were measured and compared between groups. Associations between biomarkers and symptom severity were analyzed. Serum SIRT-1 (9.36 ± 9.62 vs. 18.52 ± 14.90, *p* < 0.001), Nrf-2 (11.18 ± 10.53 vs. 22.05 ± 17.44, *p* < 0.001), and HO-1 (0.86 ± 0.95 vs. 1.89 ± 1.65, *p* < 0.001) levels were significantly lower in adults with ADHD compared to controls, whereas TNF-α levels did not differ between groups (7.49 ± 2.15 vs. 7.78 ± 2.06, *p* = 0.439). CRP levels were modestly higher (3.50 ± 1.59 vs. 2.86 ± 1.68, *p* = 0.036), and ferritin levels were lower in the ADHD group (49.78 ± 46.68 vs. 71.54 ± 63.80, *p* = 0.035). SIRT-1 (*r* = − 0.430, *p* = 0.001), Nrf-2 (*r* = − 0.446, *p* < 0.001), and HO-1 (*r* = − 0.424, *p* = 0.001) levels were negatively correlated with ADHD symptom severity. ROC analysis demonstrated moderate discriminatory performance for HO-1 (AUC = 0.797), Nrf-2 (AUC = 0.756), and SIRT-1 (AUC = 0.753). Adult ADHD was associated with reduced serum SIRT-1, Nrf-2, and HO-1 levels, pointing to alterations in redox-regulatory mechanisms. TNF-α did not differ between groups; however, broader inflammatory pathways were not comprehensively assessed. These findings support further research on oxidative stress–related regulation and its potential value for biomarker-informed clinical strategies in adult ADHD.

**Graphical abstract:**

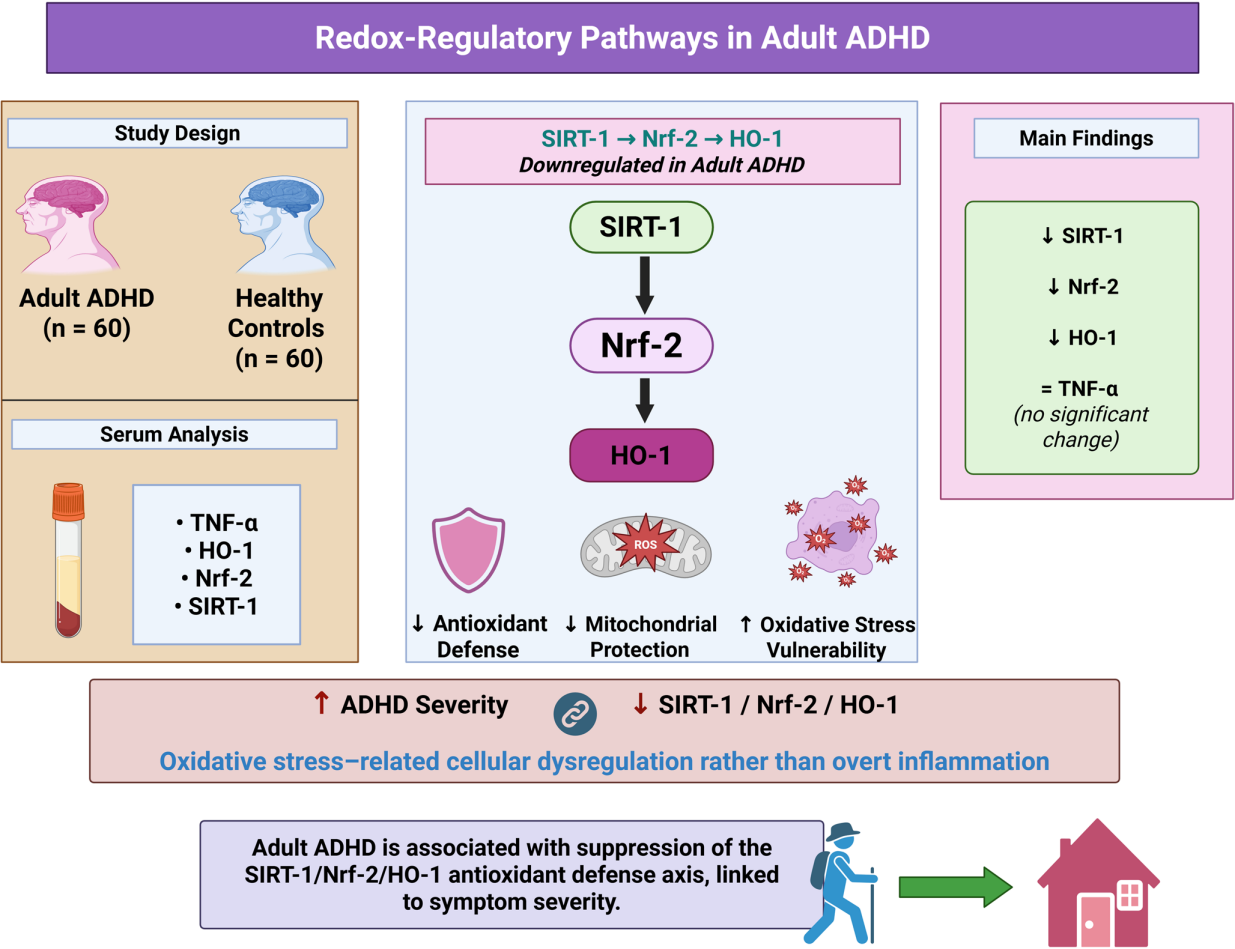

## Highlights

Adult patients with ADHD exhibit impaired antioxidant defense rather than overt peripheral inflammation.

Serum SIRT-1, Nrf-2, and HO-1 levels are significantly reduced in adults with ADHD.

TNF-α levels do not differ significantly between ADHD patients and healthy controls.

Reduced antioxidant defense markers are associated with greater ADHD symptom severity.

Dysregulation of the SIRT-1/Nrf-2/HO-1 axis may play a role in ADHD pathophysiology.

## Introduction

Attention-Deficit/Hyperactivity Disorder (ADHD) is a neurodevelopmental disorder characterized by inattention, hyperactivity, and impulsivity, typically beginning in childhood and frequently persisting into adulthood. The worldwide prevalence of ADHD in children and adolescents is reported to be 7.2%, and approximately 4% of cases are estimated to persist into adulthood. Although ADHD is considered a childhood disorder, a large proportion of affected children continue to exhibit symptoms during adolescence, and symptoms persist into adulthood in approximately 45% of cases. Due to its high prevalence, negative impact on academic, occupational, and social functioning, and frequent psychiatric and medical comorbidities, ADHD is regarded as a major public health concern (Leffa et al. 2018; Corona 2020).

The etiopathogenesis of ADHD is highly complex and multifactorial and is thought to result from the interaction of multiple biological mechanisms, including genetic susceptibility, dysregulation of neurotransmitter systems, environmental factors, neuroinflammation, and oxidative stress. Although disturbances in dopaminergic and noradrenergic systems have long been considered central to ADHD pathophysiology, accumulating evidence in recent years indicates that ADHD is not merely a neurotransmitter disorder, and that alterations in cellular stress responses and redox balance also play an important role (Joseph et al. 2015; Lopresti 2015).

Oxidative stress is defined as a state resulting from an imbalance between reactive oxygen and nitrogen species and cellular antioxidant defense mechanisms. Brain tissue is particularly vulnerable to oxidative stress due to its high oxygen consumption, high lipid content, and relatively limited antioxidant capacity. Studies investigating the role of oxidative stress in ADHD have reported heterogeneous findings regarding lipid peroxidation products, antioxidant enzymes, and total antioxidant capacity; however, the overall trend suggests increased oxidative stress and impaired antioxidant defense in ADHD (Lopresti 2015; Visternicu et al. 2024). Beyond ADHD, clinical studies in other neurodevelopmental disorders have also demonstrated alterations in Nrf2-related signaling. For example, case–control research in children with autism spectrum disorder has reported changes in NRF2, KEAP1, and GSK-3 levels, supporting the relevance of redox-regulatory pathways in neurodevelopmental conditions (Subasi Turgut et al. 2024). These findings highlight the broader clinical relevance of Nrf2-associated mechanisms in neurodevelopmental psychopathology.

One of the main reasons for this heterogeneity is the need to evaluate oxidative stress not only through damage markers but also through upstream regulatory pathways that govern cellular defense systems. Nuclear factor erythroid 2–related factor 2 (Nrf-2), heme oxygenase-1 (HO-1), and sirtuin-1 (SIRT-1) are among the key molecules that play central roles in the regulation of cellular antioxidant defense, mitochondrial function, certain metabolic pathways, and inflammatory responses (Ulu et al. 2021). Experimental models have demonstrated that SIRT-1 activates Nrf-2, thereby enhancing HO-1 expression, and that this axis simultaneously suppresses oxidative stress and inflammation. Through epigenetic regulation and modulation of transcriptional activity, SIRT-1 has been shown to influence Nrf-2 nuclear translocation and downstream antioxidant gene expression, positioning the SIRT-1/Nrf-2/HO-1 pathway as a coordinated regulatory axis rather than isolated molecular components (Yu et al. 2019; Singh and Ubaid 2020). Given the close functional interaction between redox-regulatory pathways and inflammatory signaling, tumor necrosis factor-alpha (TNF-α) was also considered relevant in this context. SIRT-1 has been shown to suppress NF-κB–mediated transcription, and Nrf-2/HO-1 activation may influence downstream cytokine expression. Therefore, simultaneous evaluation of TNF-α together with SIRT-1/Nrf-2/HO-1 may provide a more integrated understanding of the interplay between antioxidant defense mechanisms and inflammatory processes in ADHD (Singh and Ubaid 2020).

In neurodegenerative and neuropsychiatric disorders, cellular damage is thought to primarily result from oxidative stress, mitochondrial dysfunction, and an inadequate antioxidant response, a process that is largely regulated by the SIRT-1/Nrf-2/HO-1 axis (Jiao and Gong 2020; Liu et al. 2022; Sethi et al. 2025). In the context of ADHD, suppression of the Nrf-2/HO-1 and SIRT-1 axis has been suggested to reduce neuronal stress tolerance, induce mitochondrial dysfunction, and negatively affect synaptic function (Carafa et al. 2016; Mokhtar et al. 2024; Sethi et al. 2025). However, it should be noted that much of the existing evidence derives from experimental or preclinical research, and clinical data—particularly in adult ADHD populations—remain relatively limited. In contrast, findings regarding classical inflammatory cytokines, particularly tumor necrosis factor-alpha (TNF-α), have been inconsistent. Recent reviews and meta-analyses emphasize that inflammation in ADHD may represent a secondary process related to oxidative stress and impaired cellular defense rather than a primary pathogenic mechanism (Leffa et al. 2018; Alvarez-Arellano et al. 2020).

Although evidence regarding oxidative stress and antioxidant defense systems in ADHD has been steadily increasing, studies simultaneously evaluating upstream regulatory biomarkers such as TNF-α, HO-1, Nrf-2, and SIRT-1—particularly in adult ADHD populations—remain limited. Moreover, investigations examining the relationship between these biomarkers and clinical symptom severity or phenotypic characteristics of the disorder are scarce (Joseph et al. 2015; Alvarez-Arellano et al. 2020; Corona 2020). To date, only a small number of experimental studies have addressed this issue (Mokhtar et al. 2024), and comprehensive human studies integrating these regulatory markers within a single analytical framework are still lacking.

In light of these considerations, we hypothesized that adults with ADHD may exhibit dysregulation of TNF-α, HO-1, Nrf-2, and SIRT-1, along with alterations in peripheral inflammatory parameters, and that these biochemical changes may be associated with ADHD symptom severity. In this study, we aimed to compare the clinical characteristics, serum TNF-α, HO-1, Nrf-2, and SIRT-1 levels, and hematological parameters between adults with ADHD and healthy controls, as well as to evaluate the relationships between these biomarkers, symptom severity, clinical features, and laboratory findings in ADHD. A better understanding of the role of oxidative stress and cellular defense mechanisms in the pathophysiology of ADHD may contribute to the development of targeted and individualized therapeutic approaches in the future.

## Methods

### Study sample

This study was designed as a cross-sectional, single-center case–control study. The patient group consisted of 60 adults diagnosed with ADHD who presented to the Psychiatry Outpatient Clinic of Erzurum City Hospital between January 2025 and December 2025. The control group comprised 60 healthy participants without any psychiatric disorder who presented to the psychiatry outpatient clinic for consultation or for the issuance of a medical status report during the same period.

The study protocol was approved by the Scientific Research Ethics Committee of Erzurum Faculty of Medicine, University of Health Sciences (Erzurum, Türkiye) with decision number BAEK 2024/11–210, and the study was conducted in accordance with the Declaration of Helsinki. Written informed consent was obtained from all participants.

### Procedure

The study focused on the cross-sectional clinical characteristics, routine biochemical parameters, and serum fasting TNF-alpha, HO-1, Nrf-2, and SIRT-1 analyses of 120 participants.

The patient group consisted of 60 adult participants who met the diagnostic criteria for ADHD and had not received any prior treatment for ADHD. The control group consisted of 60 healthy adult participants matched to the patient group in terms of age and sex. The diagnosis of ADHD was established by a psychiatrist (NG) with at least five years of specialist clinical experience, using the Structured Clinical Interview for DSM-5 – Clinician Version (SCID-5/CV) and the Diagnostic Interview for ADHD in Adults (DIVA 2.0) (Elbir et al. 2019; Ramos-Quiroga et al. 2019; Osório et al. 2019). The presence of comorbid psychiatric disorders was excluded through a comprehensive psychiatric evaluation conducted by the same psychiatrist and the SCID-5/CV. The Adult Attention-Deficit/Hyperactivity Disorder Self-Report Scale (ASRS) was completed by all participants, and ADHD symptom severity was assessed based on ASRS scores. Clinical and sociodemographic data were recorded by the clinician. Fasting blood samples were obtained from all participants between 08:00 and 10:00. Body weight and height were measured for all participants.

Inclusion criteria for the patient group were a diagnosis of ADHD according to DSM-5 criteria, no prior treatment for ADHD, and the absence of any comorbid psychopathology other than ADHD. Additional inclusion criteria included being between 18 and 65 years of age, having no physical and/or mental disability that would interfere with completing the assessments, absence of obesity (BMI > 30 kg/m²), and providing written informed consent to participate in the study. Exclusion criteria included the presence of acute and/or chronic medical and/or autoimmune/inflammatory diseases, a history of infection at the time of sample collection or within the preceding three months, and the use of immunosuppressive, anticoagulant, or anti-inflammatory medications.

Inclusion criteria for the control group were being between 18 and 65 years of age, having no psychopathology, absence of obesity (BMI > 30 kg/m²), no physical and/or mental disability that would interfere with completing the assessments, absence of acute and/or chronic medical and/or autoimmune/inflammatory diseases, no history of infection at the time of sample collection or within the preceding three months, no use of immunosuppressive, anticoagulant, or anti-inflammatory medications, and provision of written informed consent. No changes were made to the treatment of patients who were included in the study or those who declined participation. Participants with current psychotropic medication use and/or a history of psychotropic treatment within the past six months were excluded from the study.

### Data collection instruments

#### Sociodemographic data form

This form was developed by the researchers to record demographic characteristics of the participants, including age, sex, education level, marital status, employment status, alcohol and cigarette use, height, weight, and body mass index (BMI).

#### SCID-5/CV

The Structured Clinical Interview for DSM (SCID) is one of the most widely used diagnostic tools in clinical research worldwide. The most recent version is SCID-5. SCID-5/CV is a comprehensive, standardized instrument used to assess major psychiatric disorders according to DSM-5 definitions and criteria (Osório et al. 2019). The Turkish version of SCID-5/CV has been validated and shown to be reliable (Elbir et al. 2019).

#### DIVA 2.0

DIVA was developed based on DSM-4 diagnostic criteria. It is a semi-structured diagnostic interview designed for adults, including separate criteria for inattention and hyperactivity/impulsivity in childhood and adulthood, as well as impairment in functioning resulting from symptoms (Kooij et al. 2010).

#### ASRS

The scale developed by the World Health Organization (Kessler et al. 2005) consists of two subscales: “inattention” and “hyperactivity/impulsivity.” It includes a total of 18 items, with nine items in each subscale. The items assess how frequently each symptom has occurred over the past six months. Responses are scored on a scale from 0 to 4. The Turkish validity and reliability of the scale have been established (Doğan et al. 2009), and reliability analysis demonstrated high internal consistency (Cronbach’s alpha = 0.88).ar 

All SCID-5/CV and DIVA 2.0 interviews were conducted by a board-certified psychiatrist (N.G.) with over 5 years of clinical experience in structured diagnostic assessment and adult ADHD evaluation.

### Biochemical analysis

Participants were asked to rest in a seated position between 08:00 and 10:00. Venous blood samples were drawn from the antecubital region for the analysis of HO-1, NRF-2, SIRT-1, TNF-alpha, CRP, ferritin, and hemogram parameters. Hemogram tests were processed immediately, and the results were recorded. For serum analyses, blood samples were allowed to clot at room temperature for 30 min and were then centrifuged at 3000 rpm for 10 min. CRP, TNF-alpha, and ferritin assays were performed on the obtained serum samples, and the results were documented. The remaining serum samples were aliquoted and stored at − 80 °C until ELISA analyses were conducted.

CRP, TNF-alpha, and ferritin levels were measured using spectrophotometric and immunoassay methods on the Siemens Atellica^®^ clinical chemistry analyzer (Siemens Healthineers, Erlangen, Germany). Hemogram parameters were assessed in whole blood samples collected in EDTA tubes using the Mindray^®^ hematology analyzer (Mindray BC-6800, Shenzhen, China).

Serum HO-1, NRF-2, and SIRT-1 concentrations were analyzed using ELISA kits according to the manufacturers’ standard protocols (BT Lab, Human Heme Oxygenase-1: Cat. No. E0932Hu; BT Lab, Human Nuclear Factor Erythroid 2-Related Factor 2: Cat. No. E3244Hu; BT Lab, Human Sirtuin-1: Cat. No. E2557Hu; Jiaxing Korain Biotech, Jiaxing, China) on a Rel Assay automated ELISA reader (Biobase Biodusty Co., Ltd., Jinan, China). The analytical measurement ranges were 0.1–9 ng/mL for HO-1, 0.2–60 ng/mL for NRF-2, and 0.2–60 ng/mL for SIRT-1. After confirming that the serum samples had been thawed under appropriate conditions, all analyses were performed in a single session at the Erzurum City Hospital Medical Biochemistry Laboratory.

### Statistical analysis

A priori sample size calculation was performed using G*Power software (Faul et al. 2007). Based on prior literature examining oxidative stress–related biomarkers in ADHD (Uzun Cicek et al. 2020), and assuming an effect size of 0.70, a 95% confidence level, and 90% power, a minimum of 44 participants per group (total *n* = 88) was required to detect a 10-unit difference between groups.

Statistical analyses were performed using the IBM Statistical Package for the Social Sciences (SPSS), version 22. The normality of continuous variables was assessed using the Shapiro–Wilk test, Kolmogorov–Smirnov test, Q–Q plots, skewness, and kurtosis. Descriptive statistics were presented as numbers and percentages for categorical variables and as means and standard deviations for continuous variables. The chi-square test was used to compare categorical variables. Since the assumption of normal distribution was met, the Independent Samples t-test was used for comparisons between two independent groups. Effect sizes between groups were calculated using Cohen’s d statistic. Pearson correlation analysis was applied to assess associations between two continuous variables.

Receiver operating characteristic (ROC) curve analysis was performed to calculate the sensitivity, specificity, area under the curve (AUC), and cut-off values of HO-1, Nrf-2, and SIRT-1. In addition, linear regression analysis was conducted to identify factors predicting symptom severity in patients. Statistical significance was set at *p* < 0.05.

## Results

A total of 120 participants were included in the study, consisting of 60 patients and 60 healthy controls. There were no significant differences between the patient and control groups in terms of age, sex, or BMI (*p* > 0.05). Among the 53 participants who were smokers, 27 (50.9%) were in the patient group, and among the 21 participants who reported alcohol use, 13 (61.9%) were in the patient group. The patient and control groups were similar with respect to smoking and alcohol use (*p* > 0.05). Of the 25 participants with a family history of psychiatric illness, 23 (92%) were participants with ADHD, which was significantly higher compared to the control group (*p* < 0.001). Among the ADHD participants with a family history of psychiatric illness, 14 had a diagnosis of ADHD. Only 30 (50%) of the patients in the ADHD group were employed. The sociodemographic characteristics of the participants are presented in Table 1.

**Table 1:** Comparison of Sociodemographic and Clinical Characteristics of the Patient and Control Groups

		Patient Group *n* %	Control Group *n* %	chi-square/t	*p*
Age (year)	Mean ± SD	29.98 ± 10.11	31.85 ± 7.79	1.133	0.259
BMI (kg/m^2^)	Mean ± SD	25.39 ± 3.14	25.49 ± 2.84	0.200	0.841
Gender	female	28-(46.7)	28-(46.7)		
	male	32-(53.3)	32-(53.3)		
Marital status	Married	16-(26.7)	38-(63.3)	16.401	< 0.001
	Single	40-(66.7)	20-(33.3)		
	Widowed, divorced, living apart	4-(6.6)	2-(3.4)		
Occupation	Unemployed	30-(50)	4-(6.7)	31.873	< 0.001
	Private sector employee	13-(21.7)	12-(20)		
	Public employee	17-(28.3)	44-(73.3)		
Educational status	Primary school	0-(0)	2-(3.3)	7.007	0.016
	High school	29-(48.3)	16-(26.7)		
	University	31-(51.7)	42-(70)		
Smoking	Yes	27-(45)	26-(43.3)	0.034	0.854
	No	33-(55)	34-(56.7)		
Alcohol use	Yes	13-(21.7)	8-(13.3)	0.924	0.337
	No	47-(78.3)	52-(86.7)		
Presence of mental illness in the family	Yes	23-(38.3)	2-(3.3)	20.211	< 0.001
	No	37-(61.7)	58-(96.7)		

Values are presented as mean ± standard deviation (SD) for continuous variables and number (percentage) for categorical variables. Group comparisons were performed using Independent Samples t-test for continuous variables and chi-square test for categorical variables. *p* < 0.05 was considered statistically significant. Abbreviations: BMI, body mass index

CRP levels were significantly higher (*p* = 0.036), whereas ferritin levels were significantly lower (*p* = 0.035) in patients compared with controls. Serum HO-1 (*p* < 0.001), Nrf-2 (*p* < 0.001), and SIRT-1 (*p* < 0.001) levels were also significantly lower in patients than in controls. TNF-alpha levels did not differ significantly between the groups (Table 2).

**Table 2:** Comparison of ASRS scale scores, TNF alpha, HO-1, Nrf-2, SIRT-1, and Hemogram Parameters between the Patient and Control Groups

	Patient Group *n* = 60	Control Group *n* = 60	t	*p*	effect size
	Mean ± SD	Mean ± SD			
ASRS Total Score	45.65 ± 7.39	14.57 ± 2.40	−30.986	**< 0.001**	5.66
ASRS Attention Score	23.23 ± 4.69	7.07 ± 1.40	−25.576	**< 0.001**	4.61
ASRS Hyperactivity/Impulsivity Score	22.52 ± 4.03	7.45 ± 1.70	−26.693	**< 0.001**	4.87
Leukocyte (10^9^/L)	7.11 ± 1.48	7.27 ± 1.73	0.541	0.590	0.10
Neutrophil (10^9^/L)	4.05 ± 1.34	4.18 ± 1.46	0.504	0.615	0.09
Lymphocyte (10^9^/L)	2.36 ± 0.61	2.33 ± 0.61	−0.302	0.763	0.05
Monocyte (10^9^/L)	0.53 ± 0.15	0.56 ± 0.16	1.403	0.163	0.19
Platelet (10^9^/L)	282.95 ± 69.44	277.10 ± 54.32	−0.514	0.608	0.09
CRP (mg/L)	3.50 ± 1.59	2.86 ± 1.68	−2.127	**0.036**	0.39
Ferritin	49.78 ± 46.68	71.54 ± 63.80	2.133	**0.035**	0.39
TNF alpha	7.49 ± 2.15	7.78 ± 2.06	0.776	0.439	0.14
HO-1	0.86 ± 0.95	1.89 ± 1.65	4.195	**< 0.001**	0.77
Nrf-2	11.18 ± 10.53	22.05 ± 17.44	4.133	**< 0.001**	0.75
SIRT-1	9.36 ± 9.62	18.52 ± 14.90	4.000	**< 0.001**	0.73

Data are presented as mean ± standard deviation (SD). Group comparisons were performed using Independent Samples t-test. Effect sizes were calculated using Cohen’s d (0.2-small, 0.5-medium, and 0.8-large effect size). *p* < 0.05 was considered statistically significant. Abbreviations: ASRS, Adult Attention-Deficit/Hyperactivity Disorder Self-Report Scale; CRP, C-reactive protein; TNF-α, tumor necrosis factor-alpha; HO-1, heme oxygenase-1; Nrf-2, nuclear factor erythroid 2–related factor 2; SIRT-1, sirtuin-1

In the patient group, HO-1 levels showed a moderate negative correlation with inattention (*r* = − 0.421, *p* = 0.001), hyperactivity/impulsivity (*r* = − 0.264, *p* = 0.041), and total symptom severity (*r* = − 0.424, *p* = 0.001). Similarly, Nrf-2 levels were moderately negatively correlated with inattention (*r* = − 0.434, *p* = 0.001), hyperactivity/impulsivity (*r* = − 0.292, *p* = 0.024), and total symptom severity (*r* = − 0.446, *p* < 0.001). SIRT-1 levels were also moderately negatively correlated with inattention (*r* = − 0.409, *p* = 0.001), hyperactivity/impulsivity (*r* = − 0.284, *p* = 0.028), and total symptom severity (*r* = − 0.430, *p* = 0.001). No significant associations were observed between TNF-alpha levels and clinical or laboratory parameters (*p* > 0.05) (Table 3).

**Table 3:** The relationship between TNF-alpha, HO-1, Nrf-2, and SIRT-1 levels in patients and their clinical and blood parameters

		TNF-alpha	HO-1	Nrf-2	SIRT-1
ASRS Attention Score	r	0.016	−0.421	−0.434	−0.409
	p	0.901	**0.001**	**0.001**	**0.001**
ASRS Hyperactivity/Impulsivity Score	r	0.233	−0.264	−0.292	−0.284
	p	0.073	**0.041**	**0.024**	**0.028**
ASRS Total Score	r	0.097	−0.424	−0.446	−0.430
	p	0.460	**0.001**	**0.000**	**0.001**
Age	r	−0.013	0.030	0.044	0.005
	p	0.920	0.822	0.736	0.972
BMI	r	0.021	−0.146	−0.129	−0.186
	p	0.874	0.267	0.327	0.154
Leukocyte	r	0.093	−0.002	0.020	−0.056
	p	0.481	0.988	0.879	0.672
Neutrophil	r	0.013	0.033	0.061	−0.007
	p	0.923	0.802	0.642	0.958
Lymphocyte	r	0.121	−0.049	−0.059	−0.081
	p	0.356	0.711	0.655	0.537
Monocyte	r	0.150	−0.112	−0.112	−0.089
	p	0.252	0.396	0.392	0.499
Platelet	r	−0.036	−0.038	−0.057	−0.030
	p	0.788	0.775	0.666	0.817
CRP	r	−0.118	0.049	0.035	0.085
	p	0.367	0.710	0.792	0.520
Ferritin	r	0.115	−0.076	−0.122	−0.177
	p	0.380	0.564	0.354	0.176
TNF-alpha	r	1	−0.069	−0.103	−0.053
	p		0.599	0.432	0.690
HO-1	r	−0.069	1	0.965	0.922
	p	0.599		0.000	0.000
Nrf-2	r	−0.103	0.965	1	0.932
	p	0.432	0.000		0.000
SIRT-1	r	−0.053	0.922	0.932	1
	p	0.690	0.000	0.000	

Correlation analyses between clinical variables, laboratory parameters, and serum TNF-α, HO-1, Nrf-2, and SIRT-1 levels in the patient group. Pearson correlation coefficients (r) and corresponding p values are presented. *p* < 0.05 was considered statistically significant. Abbreviations: ASRS, Adult Attention-Deficit/Hyperactivity Disorder Self-Report Scale; CRP, C-reactive protein; BMI, body mass index; TNF-α, tumor necrosis factor-alpha; HO-1, heme oxygenase-1; Nrf-2, nuclear factor erythroid 2–related factor 2; SIRT-1, sirtuin-1

**Fig. 1:**
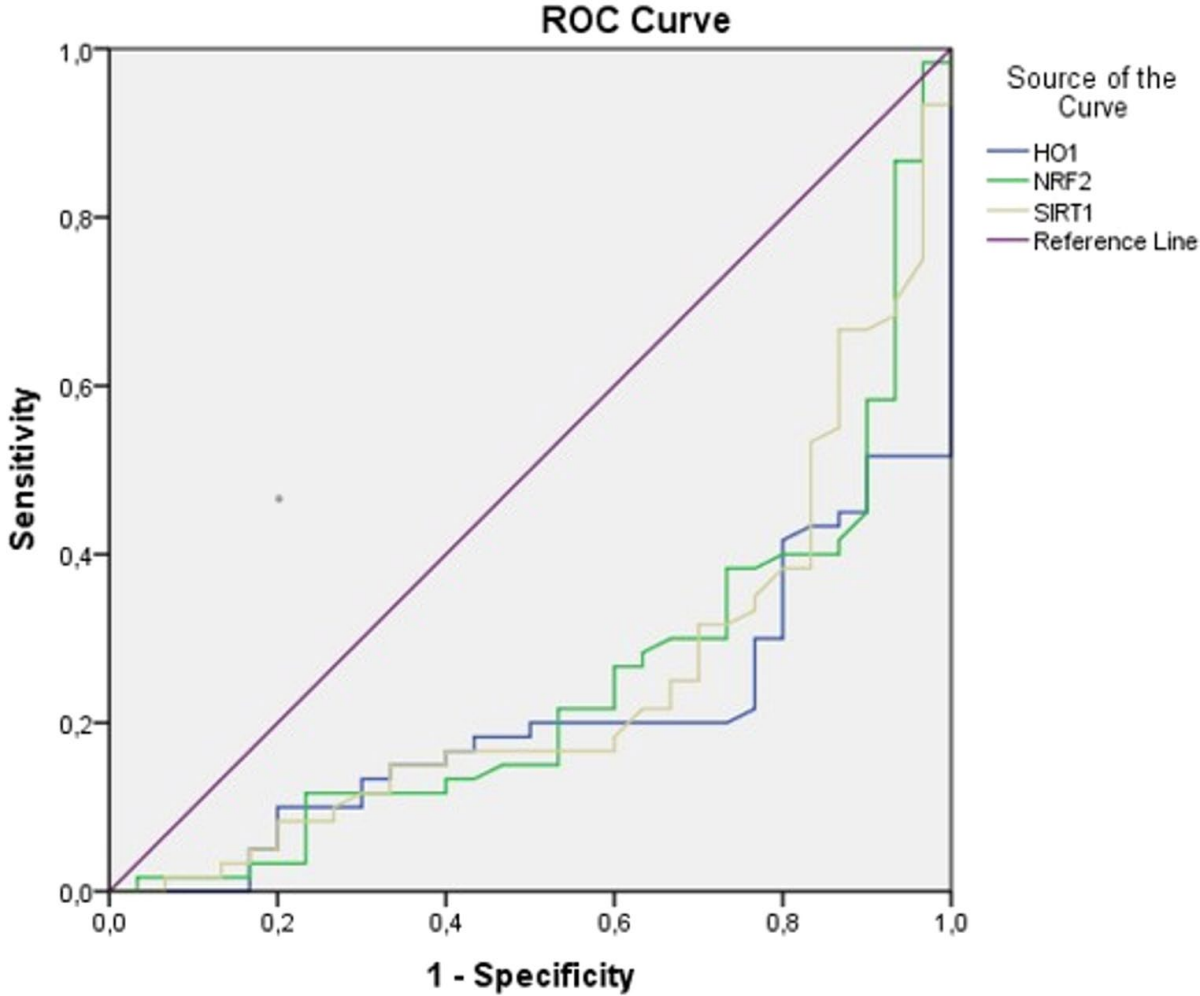
ROC Curve Analysis for HO-1, Nrf-2 and SIRT-1. HO-1 (cut-off value 0.87, sensitivity 78.3%, specificity 76.7%); Nrf-2 (cut-off value 9.8 sensitivity 70%, specificity 73.3%); SIRT-1 (cut-off value 8.3, sensitivity 75%, specificity 70%). Abbreviations: HO-1: heme oxygenases-1; Nrf-2: nuclear factor erythroid 2-related factor 2; SIRT-1: Sirtuin 1

Receiver operating characteristic (ROC) curve analysis was performed to evaluate the discriminative performance of HO-1, Nrf-2, and SIRT-1 levels in distinguishing adults with ADHD from healthy controls. The area under the curve (AUC) was 0.797 (95% CI: 0.716–0.878, *p* < 0.001) for HO-1, 0.756 (95% CI: 0.668–0.844, *p* < 0.001) for Nrf-2, and 0.753 (95% CI: 0.665–0.841, *p* < 0.001) for SIRT-1. The optimal cut-off value for HO-1 was 0.87, yielding a sensitivity of 78.3% and specificity of 76.7%. For Nrf-2, the cut-off value was 9.8 (sensitivity: 70%; specificity: 73.3%), and for SIRT-1, the cut-off value was 8.3 (sensitivity: 75%; specificity: 70%). Lower levels of HO-1, Nrf-2, and SIRT-1 were associated with increased likelihood of belonging to the ADHD group (Table 4; Fig. 1).

**Table 4:** Area Under the Curve

Test Result Variable(s)		Area	Std. Error^a^	Asymptotic Sig.^b^	Asymptotic 95% Confidence Interval	
					Lower Bound	Upper Bound
Patient group -Control group	HO-1	0.797	0.041	0.000	0.716	0.878
	Nrf-2	0.756	0.045	0.000	0.668	0.844
	SIRT-1	0.753	0.045	0.000	0.665	0.841

Receiver operating characteristic (ROC) curve analysis showing the diagnostic performance of HO-1, Nrf-2, and SIRT-1 in discriminating patients with ADHD from healthy controls. Area under the curve (AUC), standard error, asymptotic significance, and 95% confidence intervals are presented. *p* < 0.05 was considered statistically significant

a Under the nonparametric assumption

b Null hypothesis: true area = 0.5

Abbreviations: HO-1, heme oxygenase-1; Nrf-2, nuclear factor erythroid 2–related factor 2; SIRT-1, sirtuin-1

Separate hierarchical multiple regression analyses were conducted to determine the effects of independent variables on the severity of “inattention” and “hyperactivity/impulsivity” symptoms in ADHD (Tables 5, 6, 7, and 8). Independent variables (age, BMI, peripheral inflammatory parameters, TNF-alpha, HO-1, Nrf-2, and SIRT-1) were entered sequentially into the regression models with the dependent variables (ASRS-inattention and ASRS-hyperactivity/impulsivity scores). Significant associations were found between the ASRS-inattention score and age, HO-1, Nrf-2, and SIRT-1. Significant associations were also observed between the ASRS-hyperactivity/impulsivity score and HO-1, Nrf-2, and SIRT-1. Because strong positive correlations were present among HO-1, Nrf-2, and SIRT-1, only SIRT-1 was included as an independent variable in both models.

**Table 5:** Analysis results regarding the evaluation of the models

Model	*R*	*R* ^2^	Adjusted *R*^2^	Std. Error	Change Statistics				
					*R*^2^ change	F change	df1	df2	Sig. F change
1	0.348^a^	0.121	0.106	4.436	0.121	7.979	1	58	0.006
2	0.536^b^	0.287	0.262	4.031	0.166	13.270	1	57	0.001

Hierarchical multiple regression analysis showing the contribution of age and SIRT-1 to ASRS attention deficit scores in patients with ADHD. *p* < 0.05 was considered statistically significant

a Predictor: age

b Predictors: age and SIRT-1 (sirtuin-1)

**Table 6:** Results of a hierarchical multiple regression analysis for ASRS Attention Score

Model		Unstandardized Coefficients		Standardized Coefficients	t	*p*	95.0% Confidence Interval for B	
		B	Std.Error	Beta			Lower Bound	Upper Bound
1	(Constant)	28.074	1.807		15.537	0.000	24.457	31.691
	Age	−0.161	0.057	−0.348	−2.825	0.006	−0.276	−0.047
2	(Constant)	29.909	1.717		17.417	0.000	26.470	33.348
	Age	−0.161	0.052	−0.346	−3.092	0.003	−0.265	−0.057
	SIRT-1	−0.199	0.055	−0.407	−3.643	0.001	−0.308	−0.090

Results of hierarchical multiple regression analysis examining the predictors of ASRS attention deficit scores in patients with ADHD. Unstandardized coefficients (B), standardized coefficients (β), t values, p values, and 95% confidence intervals (CI) are presented. *p* < 0.05 was considered statistically significant. Abbreviations: CI, confidence interval; SIRT-1, sirtuin-1

**Table 7:** Analysis results regarding the evaluation of the models

Model	*R*	*R* ^2^	Adjusted *R*^2^	Std. Error	Change Statistics				
					*R*^2^ change	F change	df1	df2	Sig. F change
1	0.284^a^	0.081	0.065	3.894	0.081	5.089	1	58	0.028

Linear regression analysis showing the contribution of SIRT-1 to ASRS hyperactivity/impulsivity scores in patients with ADHD. *p* < 0.05 was considered statistically significant. a Predictor: SIRT-1 (sirtuin-1)

**Table 8:** Results of a lineer regression analysis for ASRS Hyperactivity/Impulsivity Score

Model		Unstandardized Coefficients		Standardized Coefficients	t	*p*	95.0% Confidence Interval for B	
		B	Std.Error	Beta			Lower Bound	Upper Bound
1	(Constant)	23.630	0.705		33.537	0.000	22.220	25.041
	SIRT-1	−0.119	0.053	−0.284	−2.256	0.028	−0.224	−0.013

Results of linear regression analysis examining the effect of SIRT-1 on ASRS hyperactivity/impulsivity scores in patients with ADHD. Unstandardized coefficients (B), standardized coefficients (β), t values, p values, and 95% confidence intervals (CI) are presented. *p* < 0.05 was considered statistically significant. Abbreviations: ASRS, Adult Attention-Deficit/Hyperactivity Disorder Self-Report Scale; CI, confidence interval; SIRT-1, sirtuin-1

A hierarchical multiple regression analysis was performed to determine the effects of age and SIRT-1 levels on inattention symptom severity in patients. The ASRS-inattention score was included as the dependent variable, and age and SIRT-1 were included as independent variables. Age was entered in the first step, and SIRT-1 was entered in the second step. Examination of the results showed that age in the first model explained 12.1% of the variance in the ASRS-inattention score. In the second model, the inclusion of SIRT-1 increased the explained variance to 28.7%. To assess how much of the total variance was explained by SIRT-1, the change in R² in the second model was examined and found to be 0.166. This indicates that SIRT-1 explained 16.6% of the variance in the ASRS-inattention score after controlling for age (R² change = 16.6; *p* = 0.001) (Table 5).

The results of the hierarchical multiple regression analysis for the ASRS-inattention score are presented in Table 6. According to the analysis shown in Table 6, both models significantly predicted the ASRS-inattention score. In the first model, age was a significant predictor of the ASRS-inattention score (β = −0.348; *p* = 0.006), indicating that increasing age was associated with lower ASRS-inattention scores. After controlling for age, SIRT-1 in the second model was also a significant predictor of the ASRS-inattention score (β = −0.407; *p* = 0.001), indicating that lower SIRT-1 levels were associated with higher ASRS-inattention scores (Table 6).

A regression analysis was conducted to determine the effect of SIRT-1 levels on hyperactivity/impulsivity symptom severity in patients. The ASRS-hyperactivity/impulsivity score was included as the dependent variable, and SIRT-1 was included as the independent variable. Examination of the results showed that SIRT-1 explained 8.1% of the variance in the ASRS-hyperactivity/impulsivity score (R² change = 8.1; *p* = 0.028) (Table 7).

The results of the regression analysis for the ASRS-hyperactivity/impulsivity score are presented in Table 8. According to the analysis shown in Table 8, the model significantly predicted the ASRS-hyperactivity/impulsivity score. Examination of the model indicated that SIRT-1 was a significant predictor of the ASRS-hyperactivity/impulsivity score (β = −0.284; *p* = 0.028), indicating that lower SIRT-1 levels were associated with higher ASRS-hyperactivity/impulsivity scores (Table 8).

## Discussion

In this study, we evaluated serum TNF-α, HO-1, Nrf-2, and SIRT-1 levels in adults with ADHD to explore the potential involvement of upstream redox-regulatory pathways. The principal findings indicate significantly lower SIRT-1, Nrf-2, and HO-1 levels in individuals with ADHD, whereas TNF-α levels did not differ between groups. In addition, reduced antioxidant defense–related biomarkers were associated with greater ADHD symptom severity.

Growing evidence suggests that oxidative stress and impaired antioxidant defense contribute to ADHD pathophysiology (Corona 2020; Visternicu et al. 2024). Experimental data indicate that inhibition of SIRT-1 suppresses Nrf-2 activation and downstream HO-1 expression (Yu et al. 2019), and loss of SIRT-1 has been associated with reduced antioxidant capacity and increased oxidative burden (Singh and Ubaid 2020). Within this framework, the concurrent reduction of SIRT-1, Nrf-2, and HO-1 observed in our study may reflect alterations within an interconnected regulatory axis rather than isolated molecular changes.

The literature addressing inflammation in ADHD remains heterogeneous (Leffa et al. 2018). While classical neurotransmitter dysregulation remains central, oxidative and nitrosative stress mechanisms have increasingly been recognized as relevant contributors (Joseph et al. 2015; Lopresti 2015). Accordingly, examining upstream regulators such as the SIRT-1/Nrf-2/HO-1 axis may offer complementary insight into cellular stress responses. Suppression of this pathway has been associated with mitochondrial dysfunction and synaptic vulnerability in neuropsychiatric conditions (Liu et al. 2022), and activation of SIRT-1 has been shown to enhance Nrf-2 nuclear translocation and HO-1 expression (Yu et al. 2019; Singh and Ubaid 2020). Dysregulation of related pathways, including the Klotho–SIRT-1–Nrf-2 axis, has also been implicated in neurodevelopmental disorders (Rana et al. 2025). These findings provide biological context for the alterations observed in our cohort.

TNF-α was included as a representative proinflammatory cytokine functionally linked to redox-regulatory mechanisms, given the regulatory interaction between SIRT-1/Nrf-2 signaling and NF-κB–mediated cytokine expression (Singh and Ubaid 2020). The absence of a significant difference in TNF-α levels aligns with prior inconsistent cytokine findings in ADHD (Leffa et al. 2018; Corona 2020). Experimental evidence suggests that activation of the Nrf-2/HO-1 pathway can modulate proinflammatory cytokines (Sethi et al. 2025), and TNF-α may function as a downstream component within a broader regulatory network (Liu et al. 2022). Taken together, these findings suggest that inflammatory processes in ADHD may vary across populations and may not be fully captured by single cytokine measurements.

Ferritin findings further contribute to this biological context. Meta-analytic and neuroimaging studies have reported reduced iron indices in ADHD (Cortese et al. 2012b; Scassellati et al. 2012), although supplementation trials have yielded inconsistent results (Cortese et al. 2012a). Given that ferritin participates in antioxidant defense by limiting iron-driven oxidative reactions (Joseph et al. 2015), reduced ferritin levels may reflect altered redox regulation rather than solely iron deficiency. Considering the role of HO-1 in heme metabolism, concurrent evaluation of ferritin and HO-1/Nrf2/SIRT-1 may offer complementary biological insight into oxidative processes in ADHD (Joseph et al. 2015).

Although TNF-α levels were similar between groups, CRP levels were modestly elevated in adults with ADHD. As a nonspecific marker of low-grade systemic inflammation, CRP may reflect subtle inflammatory activation influenced by metabolic and oxidative factors. The coexistence of reduced antioxidant biomarkers and elevated CRP may indicate a complex interplay between redox imbalance and low-grade inflammatory processes. However, given the cross-sectional design and the limited inflammatory panel assessed, these findings should be interpreted cautiously.

The observed associations between lower SIRT-1, Nrf-2, and HO-1 levels and greater symptom severity suggest that redox-regulatory alterations may have clinical correlates. Previous research has reported reduced SIRT-1 levels in children with ADHD and associations with cognitive performance (Uzun Cicek et al. 2020). While these findings may indicate biological continuity across developmental stages, longitudinal data are needed to clarify this relationship.

ROC analysis demonstrated moderate discriminative capacity for HO-1, Nrf-2, and SIRT-1 in differentiating adults with ADHD from controls. Sensitivity and specificity values in the 70–78% range suggest group-level differentiation; however, these markers do not support use as standalone diagnostic tools. Rather, they may contribute to biological profiling approaches or multimarker models in future research.

Several limitations should be considered. The cross-sectional design precludes causal inference. Self-report measures may introduce reporting bias. Dietary habits and sleep patterns, which can influence inflammatory and oxidative markers, were not systematically evaluated. Peripheral biomarker assessment reflects central processes only indirectly, and broader inflammatory panels were not assessed. Therefore, findings should be interpreted within these constraints and confirmed in longitudinal and mechanistic studies.

## Conclusion

In summary, our findings indicate that adults with ADHD exhibit lower serum levels of SIRT-1, Nrf-2, and HO-1, and that these reductions are associated with symptom severity. While the cross-sectional design does not allow causal conclusions, the observed pattern is consistent with a potential involvement of the SIRT-1/Nrf-2/HO-1 regulatory axis in oxidative stress–related processes in ADHD. The absence of significant TNF-α differences suggests that classical inflammatory markers may not uniformly reflect the underlying biological alterations in this population. Rather than serving as diagnostic markers, molecules within this axis may represent indicators of underlying biological state or vulnerability. These results underscore the importance of further longitudinal and mechanistic research to clarify the role of redox-regulatory pathways in ADHD and to explore their potential relevance for biomarker-guided and individualized therapeutic approaches.

## Data Availability

All the data generated or analysed during this study are included in this article. The data will be available upon reasonable request (contact persons: corressponding author).

## Declarations

### AI use declaration

Artificial intelligence (AI) tools were used solely to improve the academic English language quality of this manuscript. The scientific content, interpretation of findings, and all intellectual contributions were made entirely by the authors. The authors have thoroughly reviewed and approved the final version of the manuscript.
